# Expression Profiling of All Protein-Coding Genes in Wild-Type and Three DNA Repair-Deficient Substrains of *Escherichia coli* K-12

**DOI:** 10.1002/cfg.140

**Published:** 2002-02

**Authors:** Camilla Salmelin, Juhani Vilpo

**Affiliations:** 1 Leukemia Research Laboratory Department of Clinical Chemistry Tampere University Hospital and Tampere University Medical School Tampere Finland; 2 Leukemia Research Laboratory Department of Clinical Chemistry Tampere University Hospital and Tampere University Medical School P.O. Box 2000 Tampere FIN-33521 Finland

## Abstract

Gene chips or cDNA arrays of the entire set of *Escherichia coli* (*E. coli*) K12 genes were used to measure the expression, at the mRNA level, of all 4290 protein-coding genes in wild-type (WT) and three DNA repair-deficient derivative strains: (i) AB1157 (WT), (ii) LR39 (*ada, ogt*), (iii) MV1932 (*alkA1, tag-1*) and (iv) GM5555 (*mutS*). The aim was to investigate whether disruption of a single gene would result in significant deviation in the expression of other genes in these organisms. We describe here a simple approach for a stringent statistical evaluation of cDNA array data. This includes: (i) determination of intra- and interassay variation coefficients for different expression levels, (ii) rejection of biased duplicates, (iii) mathematical background determination, and (iv) comparison of expression levels of identical copies of a gene. The results demonstrated a highly significant correlation of gene expression when the mutants were individually compared with the wildtype. Altogether, 81 deviations of the expression of 59 genes were noted, out of 12,870, when 3-fold or greater up- or down-regulation was used as a criterion of differential expression. In the light of current knowledge of *E. coli* biology, the differential expression did not follow any logical pattern. In fact, the deviations may simply represent inter-assay variation. The results obtained here with a simple model organism are different from those obtained with most mammalian knockouts: disruption of the function of a single gene does not, under good growth conditions, necessarily result in great changes in the expression of other genes.


**Comparative and Functional Genomics**
*Comp Funct Genom* 2002; **3**: 3–13.
**DOI:** 10.1002/cfg.140

**Research Article**

### Expression profiling of all protein-coding genes in wild-type and three DNA repair-deficient substrains of *Escherichia coli* K-12

Camilla Salmelin and Juhani Vilpo*
*Leukemia Research Laboratory, Department of Clinical Chemistry, Tampere University Hospital and Tampere University Medical School, Tampere, Finland*

*Correspondence to:
Department of Clinical Chemistry, Tampere University Hospital, P.O. Box 2000, FIN-33521 Tampere, Finland.
E-mail: juhani.vilpo@tays.fi

Received: 13 September 2001
Accepted: 6 December 2001
Published online:
15 January 2002

### Abstract

**Gene chips or cDNA arrays of the entire set of *Escherichia coli* (*E. coli*) K12 genes were used to measure the expression, at the mRNA level, of all 4290 protein-coding genes in wild-type (WT) and three DNA repair-deficient derivative strains: (i) AB1157 (WT), (ii) LR39 (*ada*, *ogt*), (iii) MV1932 (*alkA1*, *tag-1*) and (iv) GM5555 (*mutS*). The aim was to investigate whether disruption of a single gene would result in significant deviation in the expression of other genes in these organisms. We describe here a simple approach for a stringent statistical evaluation of cDNA array data. This includes: (i) determination of intra- and interassay variation coefficients for different expression levels, (ii) rejection of biased duplicates, (iii) mathematical background determination, and (iv) comparison of expression levels of identical copies of a gene. The results demonstrated a highly significant correlation of gene expression when the mutants were individually compared with the wild-type. Altogether, 81 deviations of the expression of 59 genes were noted, out of 12,870, when 3-fold or greater up- or down-regulation was used as a criterion of differential expression. In the light of current knowledge of *E. coli* biology, the differential expression did not follow any logical pattern. In fact, the deviations may simply represent inter-assay variation. The results obtained here with a simple model organism are different from those obtained with most mammalian knockouts: disruption of the function of a single gene does not, under good growth conditions, necessarily result in great changes in the expression of other genes.** **Copyright © 2002 John Wiley & Sons, Ltd.**

**Keywords:** *Escherichia coli*; gene expression; cDNA array; DNA repair; mutant

### Introduction

Gene disruption techniques, also known as gene knockout, have proved to be important tools in assessing the functions of genes in various forms of life, ranging from bacteria to man. Knockout mice, for instance, are invaluable models for the study of mutations similar to those found in human diseases. Germ line gene disruption in mammals has resulted in unexpected phenotypes. This, on the other hand, is associated with a surprising functional redundancy of gene products as well as with an astronomic amount of possible interactions of proteins inside the cell. Mammalian cells remain very complicated systems in which to study the effect of single gene disruption on the expression and function of all other genes. The completion of the *E. coli* genome projects (Blattner *et al.*, 1997) has permitted the development of new tools for genome-wide analysis in this model organism.

We have been interested in the biological effects of a therapeutic alkylating agent, chlorambucil. Wild-type (WT) and DNA repair-deficient *E. coli* strains have served as model organisms (Salmelin *et al.* 2000). The importance of DNA repair, shown by reversal of damage and attenuation of the toxicity of chlorambucil, was indicated by the susceptibility of cells lacking direct DNA repair or $O^6$-methylguanine-DNA methyltransferase I and II (*ada*, *ogt*). Similarly, the protective role of base

Copyright © 2002 John Wiley & Sons, Ltd.

excision repair was substantiated by demonstration of even more increased susceptibility to chlorambucil among cells lacking 3-methyladenine-DNA glycosylase I and II (*alkA1*, *tag-1*). Cells deficient in mismatch repair (*mutS*) appeared to be only slightly more sensitive than normal cells to chlorambucil. These results clearly demonstrated that, dependent on the individual gene, gene knockout results in specific functional disturbance of *E. coli* cells. The traditional interpretation of this kind of outcome is straightforward: gene disruption results in paralysis of the corresponding function. In the present paper we report the assessment of another possibility, namely, that single gene disruption might cause unexpected changes in the homeostasis of the expression of genes whose involvement is not anticipated. To this end we analyzed, at the mRNA level, the expression of all protein-coding genes of these four *E. coli* strains. Specific emphasis was paid to the statistical interpretation of the results.

### Materials and methods

### *E. coli* strains and culture

The four *E. coli* strains and their respective proteins and functions have been reviewed briefly recently (Salmelin *et al*., 2000). The strains (a generous gift from Dr. Lene Rasmussen, Department of Molecular and Cellular Toxicology, Harvard School of Public Health) were (i) AB1157 (WT): *argE3 hisG4 leuB6 proA2 thr-1 ara-14 galK2 lacY1 mtl-1 xyl-1 thi-1 rpsL31 supE44 tsx-33*, (ii) LR39, as AB1157, but *ada*::Kan$^r$ *ogt*::Cm$^r$, (iii) MV1932, as AB1157, but *alkA1 tag-1*, and (iv) GM5555, as AB1157, but *mutS*::Tn*10*.

### Bacterial growth and isolation of total RNA

Samples for gene expression analyses were taken from exponentially growing cultures. It was important to assess global gene expression under conditions similar to those where the susceptibility to chlorambucil had been determined, i.e. when the cells had been permeabilized by using polymyxin B nonapeptide (PMBN), as described in detail elsewhere (Salmelin *et al.,* 2000).

The cells were cultured in Luria-Bertani (LB) nutrient medium (Sambrook *et al*., 1989). A single colony of each *E. coli* strain was used to inoculate LB medium and it was cultured at 37°C with shaking overnight. Next, 10 ml of this culture was added to a 250 ml Erlenmayer flask containing 40 ml fresh LB medium and it was incubated for 90 minutes at 37°C. After incubation, 10 µg PMBN/ml was added and the flasks were incubated for 2 hours at 37°C in a shaking incubator. This incubation time was chosen on the basis of the results of earlier experiments (Sambrook *et al*., 1989). After the second incubation the Erlenmayer flasks were placed on ice and RNA isolation was started immediately.

Total RNA from logarithmic growth-phase cells was isolated according to the protocol recommended by Sigma-Genosys. In order to minimize errors originating from RNA preparation (Arfin *et al*., 2000), two samples from each culture were preparated simultaneously and finally pooled for the cDNA array hybridization. The detailed protocol is available on the Internet at the Sigma-Genosys homepage: **http://www.genosys.com**. To remove contaminating genomic DNA from purified RNA, the samples were treated with RNase-free DNase I. The RNA was extracted again with three phenol (acidic) extractions followed by one phenol : chloroform : isoamyl alcohol (25 : 24 : 1) extraction and after that by one chloroform : isoamyl alcohol (24 : 1) extraction. RNA was precipitated (as in the protocol used for RNA isolation) and centrifuged at 12 000 x *g* for 30 minutes. The pellet was washed with 70% ethanol, re-centrifuged and dissolved in diethyl pyrocarbonate (DEPC)-treated water. The RNA pellet was stored in DEPC-treated water at -20°C after being quantified by absorbance at 260 nm. High quality of the RNA was confirmed by using agarose gel electrophoresis (1.2% agarose gel).

### Hybridization, and analysis of DNA arrays

Expression profiling was performed as described in detail elsewhere (Panorama™ *E. coli* Gene Arrays, Protocol Booklet, available at **http://www.genosys.com**). In short, after RNA isolation the procedure consists of (i) generation of $^{33}$P-labeled cDNA from the RNA samples, (ii) hybridization of labeled cDNA to duplicate arrays (Panorama™ *E. coli* Gene Arrays, Sigma-Genosys used in this work) representing 4290 PCR-amplified open reading frames, (iii) autoradiography of the arrays, and (iv) analysis of the expression patterns.

The primary data consisted of duplicate pixel intensities for all 4290 ORFs. For intra- and

Copyright © 2002 John Wiley & Sons, Ltd. 

inter-strain comparisons the data of each membrane were normalized by dividing all sampled intensities by the mean sampled intensity of all gene points (except the control points).

### Results and discussion

### Whole genome perspective

The expression profiles of the four *E. coli* stains were very similar, as indicated in the between-strain correlation analysis illustrated in Figure 1. Considerably smaller variation was observed in a within-strain comparison when duplicates of individual gene points were compared. Although the intra-strain variability in gene expression as a whole was smaller than the variability between the strains (Figure 2), this may simply represent an inter-assay effect rather than an overall difference in gene expression between the strains (see also Statistical Considerations, below). The inter-assay variation may also indicate variations between individual cDNA array membranes. This was shown to concern variable background levels of different membranes (cf Table 1 below). 'Between-slide' variation has been demonstrated by using comparative hybridization with fluorescent probes (Tseng *et al.*, 2001).

We revealed a total of 130 genes in the three mutant strains whose expression at the mRNA level differed 2-fold or more from that of the WT. Sixty-six genes differed 3-fold or more and these genes were selected for further scrutiny as described below. This decision was based on two factors: (i) we wanted to examine whether there are gross inter-strain differences between the mutants versus WT, and (ii) previous work relying on very similar cDNA array hybridization methodology resulted in the conclusion that a 2.5-fold expression difference indicates significantly different expression, with 99% confidence in the two tails of the data (Tao *et al.*, 1999).

### Statistical considerations

The raw data consisted of pixel intensities, in duplicate, corresponding to relative hybridization signals of mRNAs representing all open reading frames (ORFs) of *E. coli*. There were a total of 34 320 data points representing gene expression. Corresponding pixel intensities of background signals and known hybridization standards were also obtained. We made use of these data to calculate the reliability of low values approaching background, and analytical precision at different levels of gene expression. This information was used to re-evaluate the reliability of the basic procedure. The primary selection concerned all genes that showed 3-fold or greater differences of expression when compared with the wild-type. Originally, 66 such genes were found.

The background values for each strain were determined from 22 dedicated replicate array points. The pixel intensities of background values were transformed to percentages of whole genome

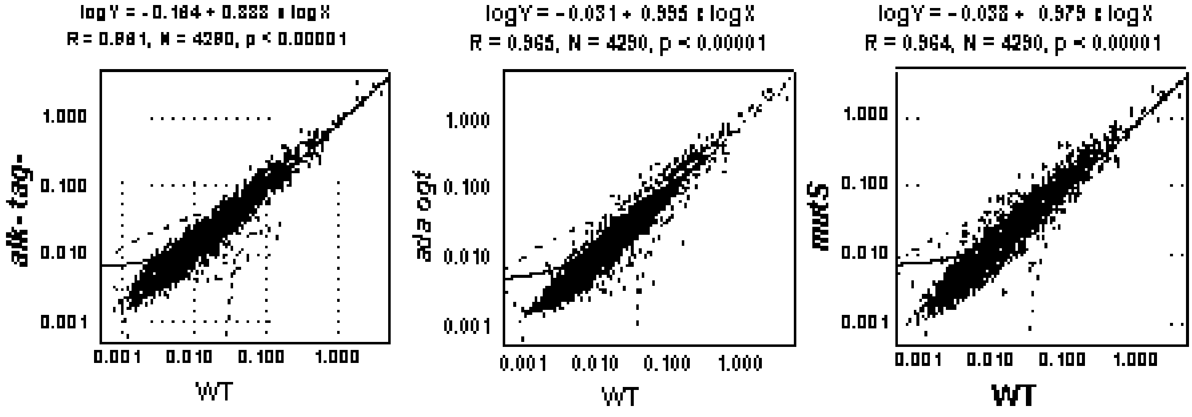

**Figure 1.** Gene expression in three DNA repair-deficient *E. coli* strains compared with WT. Least squares regression was computed according to the Pearson product-moment correlation method after logarithmic transformation of the data representing percentage expression of each individual gene. Confidence intervals were computed to correspond with 95% limits. The percentage transformation was carried out with normalized data

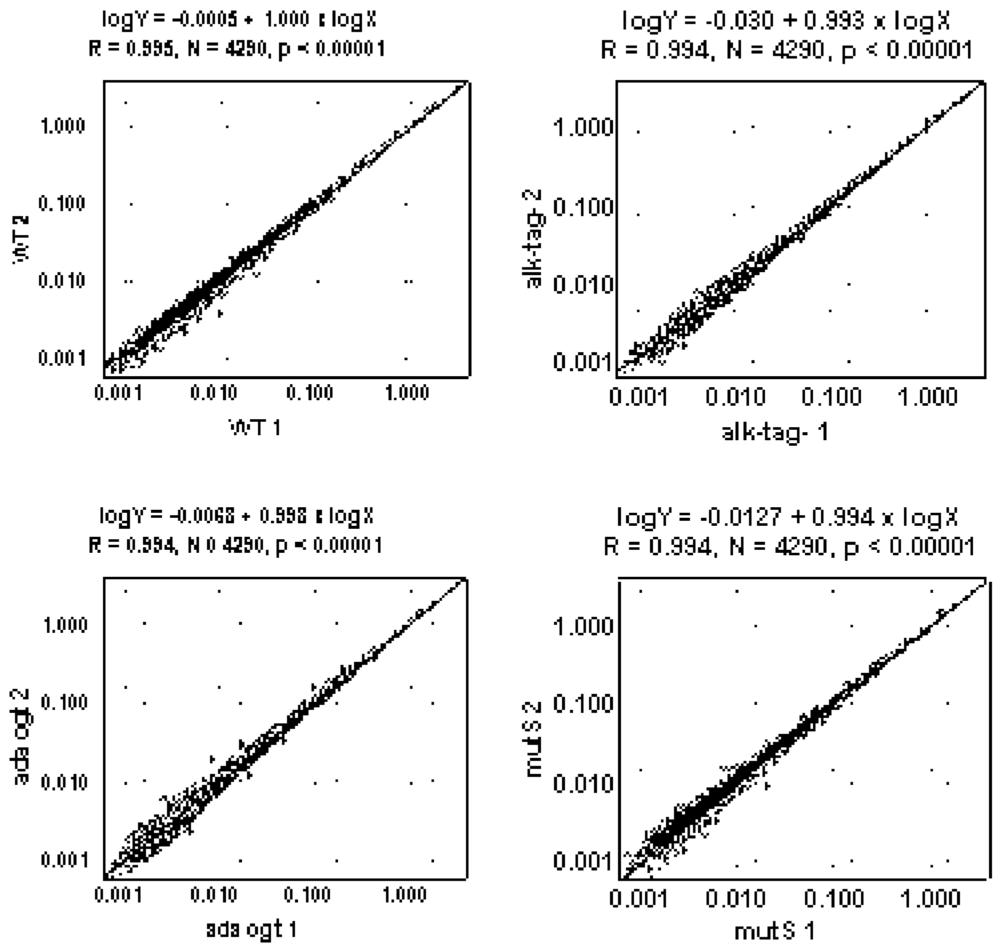

**Figure 2.** Correlations of duplicate analyses of the expression of all ORFs in WT and three DNA repair-deficient *E. coli* strains. Least squares regression was computed according to the Pearson product-moment correlation method after logarithmic transformation of the data representing percentage expression of each individual gene. Confidence intervals were computed to correspond with 95% limits (obscured by data points). The percentage transformation was carried out with normalized data expression. These figures and their statistical treatment were used to determine the sensitivity of the assay (Table 1). We chose an arbitrary detection limit such that 99.7% of all possible background values remained below this level. According to this sensitivity rule, the expression of eight out of 66 selected genes fell below the sensitivity level. Fold expressions of these genes were changed accordingly and two of these eight genes did not thereafter satisfy the '3-fold' rule. These were *b4273* (*yi22_6*) and *gatA* (see Table 2B).

The other statistical concern was the precision of the method. This information is necessary for validation of individual data points. The practical question was, how large is the maximal acceptable bias between duplicates? Duplicate assays provide a very convenient tool to determine intra-assay precision. To this end, we used the formula

$$SD = \sqrt{\sum d^2/N}$$

where SD = standard deviation, $d$ = difference between duplicates and $N$ = total number of determinations (Reed and Henry, 1974). We determined the SD values for the three different expression ranges of all four strains examined. This allowed us to determine the coefficients of variation (CVs)

Table 1. Background values for four *E. coli* strains in the global analysis of 4290 ORFs at the mRNA level[a]

| Parameters | Strains AB1157 | MV1932 | LR39 | GM5555 |
|---|---|---|---|---|
| Mean | 0.95 | 1.57 | 1.63 | 1.31 |
| Median | 0.92 | 1.58 | 1.46 | 1.31 |
| SD | 0.22 | 0.26 | 0.62 | 0.16 |
| Minimum | 0.61 | 1.16 | 1.17 | 0.11 |
| Maximum | 1.45 | 2.28 | 3.52 | 1.72 |
| 25% | 0.078 | 1.41 | 1.34 | 1.20 |
| 75% | 0.098 | 1.69 | 1.61 | 1.41 |
| Sensitivity(mean + 3 SDs) | 1.61 | 2.35 | 3.49 | 1.79 |

[a]The figures represent the statistics for 22 averages of 44 pre-selected control (blank) points in duplicate. The pixel numbers were first normalized to represent a percentage value of all genes plus control points (100%). To facilitate comparison, the percentage values were multiplied by 100 for this Table. For instance, the first figure 0.95 means that the average background for the AB1157 strain analysis was 0.0095% of the total expression (100%). The sensitivity limit represents the upper 99.7% of all possible background values.

for the different expression levels. Very similar intra-assay variations were observed (Table 3). As shown below, this information was applied to the acceptance or rejection of values represented by biased duplicates. Two recent analysis of global gene expression in *E. coli* did not take this opportunity into account (Tao *et al.*, 1999; Arfin *et al.*, 2000). Unfortunately, most published investigations do not give pertinent analytical variations. However, as shown here, random errors occur and must be corrected or data eliminated.

We arbitrarily chose to accept the duplicates if they were within $\pm$ 3 SDs of the average of the two values. This decision meant that all values within a 99.7% confidence interval would be accepted for further analysis. The procedure resulted in rejection of four of the selected 66 genes, as indicated in Table 2: *yhiE* in AB1157 and in LR39, *cysB* in LR39, *pnhA* in GM5555, and *b2640* in GM5555. This kind of validation process is possible only if duplicate cDNA arrays are used, or corresponding information of assay precision is otherwise available.

We estimated analytical precision also on the basis of comparison of gene expression in the wild-type and each of the three mutant strains. In this case, a mean value of each duplicate determination was used as illustrated in Figure 1. The SD values were calculated according to Reed and Henry (1974; equation 1). This allowed us to determine CVs for the different expression levels (Table 4). It shall be emphasized that this variation corresponds to the total variation (intra-assay, inter-assay and inter-strain). The use of duplicate means, on the other hand, results in a small underestimation of this total variation.

Another way to look at the global genomic data, not previously used, is to compare the results concerning mRNAs transcribed from identical copies of one gene. The cDNA of a gene should hybridize to all copies of the gene in a similar way. Examples are as follows: 6 identical copies of the *insA*-gene (*insA1* – *insA6*) yielded the following fold expressions (MV1932/ WT): 1.4, 1.3, 1.4, 1.3, 1.3, 1.2. (LR39/ WT): 1.4, 1.5, 1.6, 1.5, 1.5, 1.2. (GM5555/ WT): 1.4, 1.5, 1.6, 1.4, 1.4, 1.1. The current assay system should not make any distinction between these similar mRNA species. This indicates a good assay precision, but deviations were also found. For instance, a similar comparison with 6 identical copies of the *yi22*-gene yielded less precise results in the strain LR39: (LR39/WT): -1.8, -2.9, -1.9, -1,8, -3.5, -3.5 compared to (MV1932/WT): 2.1, 2.2, 1.6, 1.9, 2.1, 1.7 and (GM5555/WT): -1.3, -1.3, -1.6, -1.2, -1.6, -1.7. Hence, the expression analysis of *yi22* remains insufficient in this case. In Table 5 some examples of these genes and their expression are shown. This approach led to rejection of two genes of the 66 selected genes, namely *yi22_5* and *b4273* (*yi22_6*). The gene *b4273* (*yi22_6*) is now rejected by two different criteria (see above). We recommend the application of this quality control approach in all cases where more than one copy of the pertinent gene is active.

### Functional groups and protein functions of differentially expressed genes

The final number of selected genes having 3-fold or greater expression differences in mutant strains compared with WT was 59/(3 × 4290). In other words, only 0.46% of the genes in the three mutants showed a 3-fold or greater difference compared with the WT. The functional groups, protein products, and fold differences of these genes are given in Table 4. Among these 59 genes, the expression of 40 was changed in one strain, that of 16 in two strains and only three were different from the wild type in all three mutant strains. The differentially expressed genes were evenly distributed among the 19 functional groups (Table 6). Furthermore, the three mutants had similar amounts of differentially

Copyright © 2002 John Wiley & Sons, Ltd. 

Table 2. Pixel intensities of duplicates and fold differences in expression of genes, initially selected on the basis of their expression being at least 3-fold compared with wild-type. The pixel levels had not been normalized between different hybridization arrays in this phase. (A) AB1157 (WT) versus MV1932. (B) AB1157 (WT) versus LR39. (C) AB1157 (WT) versus GM5555

**(A)**

| Gene name | Pixels AB1157 | | Pixels MV1932 | | Fold[a] |
|---|---|---|---|---|---|
| b0465 | 1405 | 1458 | 2139 | 2165 | 3.0 |
| b1374 | 7951 | 7470 | 1373 | 1120 | −3.0 |
| b1375 | 20852 | 20895 | 2937 | 2318 | −5.2 |
| b1544 | 20434 | 20138 | 2155 | 1517 | −7.2 |
| b1795 | 20975 | 24300 | 1815 | 2367 | −7.1 |
| b3913 | 14888 | 14832 | 2653 | 2417 | −3.8 |
| b3914 | 38773 | 36549 | 6843 | 6633 | −3.6 |
| *cspA* | 106675 | 107571 | 8018 | 9502 | −6.0 |
| *cspB* | 97906 | 107442 | 5508 | 5341 | −9.3 |
| *cspG* | 15593 | 15652 | 1408 | 1303 | −5.7 |
| *dps* | 23684 | 23567 | 1622 | 1550 | −7.3 |
| *dsbB* | 1564 | 1728 | 2264 | 2787 | 3.1 |
| *ebgA* | 2431 | 2065 | 3691 | 3559 | 3.3 |
| *ftn* | 5622 | 5601 | 9909 | 10310 | 3.7 |
| *gadA* | 61603 | 61799 | 6674 | 6360 | −4.5 |
| *gadB* | 83618 | 74947 | 6985 | 7371 | −5.3 |
| *galT* | 39468 | 38854 | 2794 | 2306 | −7.6 |
| *osmY* | 19337 | 17680 | 2499 | 3340 | −3.1 |
| *proA* | 3199 | 3302 | 4737 | 5239 | 3.2 |
| *rhsB* | 8538 | 8856 | 1335 | 1500 | −4.0 |
| *yhiX* | 13657 | 13801 | 1376 | 1472 | −4.8 |

**(B)**

| Gene name | Pixels AB1157 | | Pixels LR39 | | Fold[a] | Comments[b] |
|---|---|---|---|---|---|---|
| b0329 | 10956 | 11869 | 2104 | 2440 | −3.1 | |
| b1374 | 7951 | 7470 | 1369 | 1243 | −3.8 | F = −3.3 |
| b1375 | 20852 | 20895 | 1799 | 1968 | −8.1 | |
| b1419 | 7657 | 8936 | 2397 | 2464 | −3.0 | |
| b1544 | 20434 | 20138 | 984 | 1435 | −12.3 | > 3 SD<br>F = −10.6 |
| b1551 | 7010 | 7665 | 1288 | 1183 | −4.3 | F = −3.8 |
| b1552 | 9125 | 8528 | 1951 | 1871 | −3.4 | |
| b1675 | 2697 | 2389 | 6458 | 5990 | 3.3 | |
| b1826 | 1255 | 1207 | 2669 | 2766 | 3.0 | |
| b4273 | 5187 | 5402 | 1032 | 938 | −3.5 | GC<br>F = −2.3 |
| *cspA* | 106675 | 107571 | 21864 | 22955 | −3.1 | |
| *cspB* | 97906 | 107442 | 6864 | 6408 | −10.0 | |
| *cspD* | 12863 | 11900 | 23768 | 25247 | 3.1 | |
| *cspG* | 15593 | 15652 | 1627 | 1285 | −6.9 | F = −6.7 |
| *cyoC* | 8050 | 8571 | 1712 | 1921 | −3.0 | |
| *cysB* | 17924 | 16974 | 3446 | 2571 | −3.5 | > 3 SD |
| *dppC* | 42615 | 43324 | 93627 | 90560 | 3.3 | |
| *fimI* | 19708 | 18784 | 2996 | 3478 | −3.8 | |
| *galT* | 39468 | 38854 | 1868 | 1772 | −13.9 | |
| *gatA* | 4685 | 5362 | 1087 | 997 | −3.1 | F = −2.1 |
| *gltA* | 37465 | 37595 | 4776 | 4469 | −5.2 | |
| *himA* | 7542 | 6990 | 13755 | 15452 | 3.3 | |
| *hycF* | 26047 | 26657 | 58450 | 61829 | 3.5 | |
| *icdA* | 47893 | 47503 | 7944 | 7934 | −3.9 | |
| *katE* | 14869 | 14732 | 2873 | 2626 | −3.5 | |
| *melB* | 20636 | 18037 | 3209 | 3542 | −3.7 | |
| *oppA* | 376884 | 360422 | 76431 | 67116 | −3.3 | |

Copyright © 2002 John Wiley & Sons, Ltd. 

Table 2. Continued

**(B)**

| Gene name | Pixels AB1157 | | Pixels LR39 | | Fold[a] | Comments[b] |
|---|---|---|---|---|---|---|
| *melB* | 20636 | 18037 | 3209 | 3542 | −3.7 | |
| *oppA* | 376884 | 360422 | 76431 | 67116 | −3.3 | |
| *rhsA* | 5282 | 6699 | 1266 | 1314 | −3.4 | F = −3.1 |
| *rhsB* | 8538 | 8856 | 963 | 1222 | −5.8 | F = −4.5 |
| *rpmE* | 37276 | 37107 | 79147 | 77009 | 3.4 | |
| *thiH* | 63352 | 63262 | 13093 | 12665 | −3.0 | |
| *ybgE* | 4731 | 4850 | 9152 | 8946 | 3.1 | |
| *yciD* | 4207 | 3598 | 10845 | 11140 | 4.4 | |
| *ydaC* | 1152 | 1138 | 3054 | 3132 | 3.7 | |
| *yfeC* | 10261 | 11107 | 27507 | 29246 | 3.6 | |
| *ygfE* | 22060 | 21575 | 4625 | 5052 | −3.3 | |
| *yheE* | 8375 | 8525 | 29657 | 29676 | 4.8 | |
| *yhiE* | 23348 | 12109 | 3147 | 4997 | −3.2 | > 3 SD |
| *yhiX* | 13657 | 13801 | 1727 | 1925 | −4.9 | |
| *yi22_5* | 17850 | 17723 | 3600 | 3048 | −3.5 | GC |
| *yqjF* | 4803 | 4865 | 11000 | 11762 | 3.2 | |

**(C)**

| Gene name | Pixels AB1157 | | Pixels GM5555 | | Fold[a] | Comments[b] |
|---|---|---|---|---|---|---|
| b0753 | 10428 | 10141 | 30704 | 30735 | 4.3 | |
| b1005 | 11839 | 14252 | 26884 | 28543 | 3.1 | |
| b1375 | 20852 | 20895 | 3088 | 3398 | −5.2 | |
| b1544 | 20434 | 20138 | 2817 | 2630 | −6.0 | |
| b1675 | 2697 | 2389 | 8512 | 9183 | 4.3 | |
| b1826 | 1255 | 1207 | 3588 | 3724 | 3.7 | |
| b2640 | 2283 | 2522 | 7097 | 10539 | 4.5 | > 3 SD |
| b3047 | 2706 | 2887 | 6900 | 6903 | 3.5 | |
| b3975 | 2426 | 2899 | 11328 | 12228 | 5.5 | |
| *fliC* | 5781 | 6184 | 17999 | 17446 | 4.2 | |
| *gadA* | 61603 | 61799 | 178910 | 174069 | 4.1 | |
| *gadB* | 83618 | 74947 | 179769 | 174787 | 3.2 | |
| *gapC_1* | 8503 | 9434 | 29605 | 30425 | 4.8 | |
| *gapC_2* | 6997 | 7288 | 28058 | 27404 | 5.5 | |
| *gltA* | 37465 | 37595 | 8444 | 8452 | −3.1 | |
| *hycF* | 26047 | 26657 | 66039 | 63623 | 3.5 | |
| *phnA* | 4048 | 3857 | 14088 | 6167 | 3.2 | > 3 SD |
| *rho* | 17832 | 18675 | 63873 | 63611 | 5.0 | |
| *rhsB* | 8538 | 8856 | 1357 | 1236 | −5.4 | |
| *rpmE* | 37276 | 37107 | 85450 | 88405 | 3.4 | |
| *sdhD* | 16569 | 15864 | 42117 | 31032 | 3.2 | |
| *thiH* | 63352 | 63262 | 12642 | 11140 | −3.7 | |
| *yccJ* | 10959 | 11151 | 25170 | 26524 | 3.4 | |
| *ydaC* | 1152 | 1138 | 5285 | 6654 | 6.4 | |
| *yehI* | 335524 | 323879 | 85312 | 85781 | −3.1 | |
| *yfeC* | 10261 | 11107 | 33393 | 30886 | 3.7 | |
| *yheE* | 8375 | 8525 | 30641 | 29870 | 4.4 | |
| *yqjF* | 4803 | 4865 | 13689 | 12070 | 3.3 | |

[a]The primary fold values were obtained by comparing the normalized percentage expression of the mutant *E. coli* strains with those of the WT. Negative figures are used if the mutant showed lower expression than the WT. Signals that were higher in wild-type control cells (transcription repressed) were used in the numerator of the ratio, which was then converted to negative values.

[b]Fold values were changed in the indicated cases, since the values were below the accepted sensitivity of the assay. F = the new fold value based on acceptance of the sensitivity limit for the lowest acceptable value. On this basis, one gene (*gatA*) was rejected (Table 2B). The gene is marked '> 3 SD' and the pixel value is in italics if the bias between the duplicate values exceeded ±3 SDs. The item was rejected unless the values fell below the sensitivity limit and unless the acceptance of the sensitivity limit as a lower value still resulted in a difference of 3-fold or more. Gene copies producing the same protein, but showing different expression from the other copies are marked 'GC'. On this basis two genes (b4273 and yi22_5) were rejected.

Copyright © 2002 John Wiley & Sons, Ltd. 

Table 3. The intra-strain coefficients of variation (CVs)[a]

| | Coefficient of variation | | | |
|---|---|---|---|---|
| | Strain | | | |
| Expression level | wild-type | alkA tag-1 | ada ogt | mutS |
| Low < 0.01 % of total | 9.9% | 12.5% | 12.2% | 11.6% |
| Middle 0.01-0.099% of total | 7.0% | 7.2% | 9.1% | 7.6% |
| High ⩾ 0.1% | 8.2% | 6.0% | 6.3% | 7.5% |

[a]CVs were determined on the bases of duplicate observations according to Reed and Henry (1974) as described in Results and Discussion (Statistical considerations).

expressed genes. MV1932 had the lowest proportion (24%) of changes in gene expression, LR39 had the highest (43%), and GM5555 was in between (31%), when the wild-type was used as the expression reference. Of the 81 deviations of the 59 genes 38 were up-regulated (MV1932 13%, LR39 34% and GM5555 53%) and 43 down-regulated (MV1932 37%, LR39 51% and GM5555 12%).

### Are the differences between the strains real?

The statistical approach revealed several significant gene expression differences between the four *E. coli* strains analyzed. However, none of these indicated a biological compensation of the primary gene defect. The other approach to the question of expression differences between the strains was to investigate the functions of the differentially expressed genes and to attempt to link this information to the possible consequences of the original disrupted gene function. In particular, differentially expressed operons would be valuable in this regard. It was not possible, however, to link any of the currently known functions, whether up- or down-regulated, to the conceivable consequences of the original disruption of the DNA repair genes in these three mutant *E. coli* strains. Some similarities in the mutants were noted, such as relatively low expression of the cold-shock proteins CspA and CspB in MV1932 and LR39. Furthermore *b1375 (ynaE)*, *b1544 (ydfK)* and *rhsB* showed low expression in all three mutants, but lack of information on the cellular functions of these proteins does not allow further interpretation of this observation. Furthermore, we cannot exclude the possibility that the genes undergoing 3 fold or greater differential expression in different mutant strains would have functions, in addition to the currently known ones, which could compensate for the functions of the deleted genes. It remains to be studied whether better compensation mechanisms exist in other knock-out strains or under more stressful conditions.

Table 4. Inter-strain coefficients of variation (CVs)[a]

| | Coefficient of variation | | |
|---|---|---|---|
| | Strains | | |
| Expression level | alk- tag- and WT | ada ogt and WT | mutS and WT |
| Low < 0.01% of total | 47% | 32% | 39% |
| Middle 0.01–0.099% of total | 36% | 40% | 43% |
| High ⩾ 0.1% | 66% | 62% | 72% |

[a]CVs were determined on the bases of duplicate observations according to Reed and Henry (1974) as described in Results and Discussion (Statistical considerations)

Table 5. Examples of the fold induction of some groups of identical copies of a gene (*cpxP*, *insA*, *insB*, *tra8* and *yi22*). All copies have the same end product (protein)

| | Fold induction | | |
|---|---|---|---|
| Gene | MV1932/WT | LR39/WT | GM5555/WT |
| *cpxP (b3913)* | −3.8 | 1.1 | 1.0 |
| *cpxP (b3914)* | −3.6 | 1.2 | 1.3 |
| *insA_1* | 1.4 | 1.4 | 1.4 |
| *insA_2* | 1.3 | 1.5 | 1.5 |
| *insA_3* | 1.4 | 1.6 | 1.6 |
| *insA_5* | 1.3 | 1.5 | 1.4 |
| *insA_6* | 1.3 | 1.5 | 1.4 |
| *insA_7* | 1.2 | 1.2 | 1.1 |
| *insB_1* | 1.4 | 1.4 | −1.6 |
| *insB_2* | 1.4 | 1.4 | −1.4 |
| *insB_3* | 1.4 | 1.4 | −1.5 |
| *insB_4* | 1.5 | 1.3 | −1.6 |
| *insB_5* | 1.6 | 1.6 | −1.6 |
| *insB_6* | 1.4 | 1.2 | −1.7 |
| *tra8_1* | 2.1 | −1.1 | 1.1 |
| *tra8_2* | 2.6 | 1.1 | 1.1 |
| *tra8_3* | 2.6 | −1.1 | 1.1 |
| *yi22_1* | 2.1 | −1.8 | −1.3 |
| *yi22_2* | 2.2 | −2.9 | −1.3 |
| *yi22_3* | 1.6 | −1.9 | −1.6 |
| *yi22_4* | 1.9 | −1.8 | −1.2 |
| *yi22_5* | 2.1 | −3.5 | −1.6 |
| *yi22_6 (b4273)* | 1.7 | −3.5 | −1.7 |

In conclusion, in spite of individual deviations from the common expression patterns of many genes in these four *E. coli* strains, no systematic

Copyright © 2002 John Wiley & Sons, Ltd. 

Table 6. Expression ratios, functional groups and protein functions of the genes whose expression differed ⩾ 3-fold from that of the wild-type

| Functional group[a] | Gene name | Gene product | Expression in wt (% of total)[b] | Fold change as compared to the WT | | |
|---|---|---|---|---|---|---|
| | | | | MV1932 | LR39 | GM5555 |
| Carrier (13; 2.6%) | ftn | Cytoplasmic ferritin (an iron storage protein) | 0.84 | 3.7 | | |
| Enzyme (952; 0.63%) | cyoC | Cytochrome o ubiquinol oxidase subunit III | 1.24 | | −3.0 | |
| | dsbB | Reoxidizes DsbA protein following formation of disulfide bond in P-ring of flagella | 0.25 | 3.1 | | |
| | ebgA | Evolved beta-D-galactosidase, alpha subunit; cryptic gene | 0.34 | 3.3 | | |
| | gadA | Glutamate decarboxylase isozyme | 9.03 | −4.5 | | 4.1 |
| | gadB | Putative arylsulfatase regulator | 11.6 | −5.3 | | 3.2 |
| | gapC_1 | Glyceraldehyde 3-phosphate dehydrogenase C (first fragment) | 1.34 | | | 4.8 |
| | gapC_2 | Glyceraldehyde 3-phosphate dehydrogenase (second fragment) | 1.07 | | | 5.5 |
| | galT | Galactose-1-phosphate uridylyltransferase | 5.86 | −7.6 | −13.9 | |
| | gltA | Citrate synthase | 5.62 | | −5.2 | |
| | icdA | Isocitrate dehydrogenase, specific for NADP+ | 7.14 | | −3.9 | |
| | katE | Catalase; hydroperoxidase HPII(III) | 2.22 | | −3.5 | |
| | proA | Gamma-glutamylphosphate reductase | 0.48 | 3.2 | | |
| | sdhD | Succinate dehydrogenase, hydrophobic subunit | 2.43 | | | 3.2 |
| | thiH | Thiamin biosynthesis, thiazole moiety | 9.27 | | −3.0 | −3.7 |
| Factor (71; 0.93%) | himA | Integration host factor (IHF), alpha subunit; site specific recombination | 1.06 | | 3.3 | |
| | rho | Transcription termination factor Rho; polarity suppressor | 2.67 | | | 5.0 |
| IS, phage, Tn (80; 1.25%) | b1374 | Putative transposon resolvase | 2.14 | −3.0 | −3.3 | |
| Leader (12) | | | None | | | |
| Membrane (42) | | | None | | | |
| Orf (1402; 0.76%) | b0329 | Hypothetical protein | 1.67 | | −3.1 | |
| | b0465 | Putative alpha helix protein | 0.21 | 3.0 | | |
| | b1005 | Hypothetical protein | 1.91 | | | 3.1 |
| | b1375 | Hypothetical protein | 3.80 | −5.2 | −8.1 | −5.2 |
| | b1419 | Hypothetical protein | 1.51 | | −3.0 | |
| | b1544 | Hypothetical protein | 3.69 | −7.2 | −10.6 | −6.0 |
| | b1551 | Hypothetical protein | 1.33 | | −3.8 | |
| | b1675 | Hypothetical protein | 0.46 | | 3.3 | 4.3 |
| | b1795 | Hypothetical protein | 4.12 | −7.1 | | |
| | b1826 | Hypothetical protein | 0.22 | | 3.0 | 3.7 |
| | b3913 | Hypothetical protein | 2.70 | −3.8 | | |
| | b3914 | Hypothetical protein | 6.85 | −3.6 | | |
| | b3975 | Hypothetical protein | 0.48 | | | 5.5 |
| | rhsA | rhsA protein in rhs element | 1.09 | | −3.1 | |
| | rhsB | rhsB protein in rhs element | 1.58 | −4.0 | −4.5 | −5.4 |
| | ybgE | Hypothetical protein | 0.70 | | 3.1 | |
| | yccJ | Hypothetical protein | 1.62 | | | 3.4 |
| | ydaC | Hypothetical protein | 0.21 | | 3.7 | 6.4 |
| | yfeC | Hypothetical protein | 1.94 | | 3.6 | 3.7 |
| | ygfE | Hypothetical protein | 3.97 | | −3.3 | |
| | yqjF | Hypothetical protein | 0.88 | | 3.2 | 3.3 |
| Phenotype (115; 1.45%) | b1552 | Cold shock-like protein | 1.61 | | −3.4 | |
| | cspB | Cold shock protein; may affect transcription | 15.4 | −9.3 | −10.0 | |
| | cpsD | Cold shock protein | 1.85 | | 3.1 | |
| | cspG | Homolog of Salmonella cold shock protein | 2.34 | −3.1 | | |
| | osmY | Hyperosmotically inducible periplasmic protein | 2.77 | −3.1 | | |

Copyright © 2002 John Wiley & Sons, Ltd.

Table 6. Continued

| Functional group[a] | Gene name | Gene product | Expression in wt (% of total)[b] | Fold change as compared to the WT | | |
|---|---|---|---|---|---|---|
| | | | | MV1932 | LR39 | GM5555 |
| Putative carrier (8) | | | None | | | |
| Putative enzyme (472; 0.14%) | hycF | Probable iron-sulfur protein of hydrogenase 3 (part of FHL complex) | 3.94 | | 3.5 | 3.5 |
| Putative factor (57) | | | None | | | |
| Putative membrane (59; 1.14%) | b3047 | Putative membrane protein | 0.42 | | | 3.5 |
| | yciD | Putative outer membrane protein | 0.58 | | 4.4 | |
| Putative regulator (163; 0.82%) | b0753 | Putative homeobox protein | 1.51 | | | 4.3 |
| | yehI | Putative regulator | 60.0 | | | −3.1 |
| | yhiX | Putative ARAC-type regulatory protein | 2.05 | −4.8 | −4.9 | |
| Putative RNA (4) | | | None | | | |
| Putative structure (51) | | | None | | | |
| Putative transport (288; 0.23%) | yheE | Putative general secretion pathway for protein export | 1.54 | | 4.8 | 4.4 |
| Regulator (183; 0.55%) | cspA | Cold shock protein 7.4, transcriptional activator of hns | 16.0 | −6.0 | −3.1 | |
| | dps | Global regulator, starvation conditions | 3.54 | −7.3 | | |
| Structural component (91; 1.46%) | fimI | Fimbrial protein | 2.88 | | −3.8 | |
| | fliC | Flagellar biosynthesis; flagellin, filament structural protein | 0.90 | | | 4.2 |
| | rpmE | 50S ribosomal subunit protein L31 | 5.46 | | 3.4 | 3.4 |
| Transport (227; 0.44%) | dppC | Dipeptide transport system permease protein 2 | 6.43 | | 3.3 | |
| | melB | Melibiose permease II | 2.89 | | −3.7 | |
| | oppA | Oligopeptide transport; periplasmic binding protein | 55.2 | | −3.3 | |
| Total (1.38%) | 59/4290 genes | | 2.9 % of WT total mRNA | 21 | 35 | 25 |

[a]The revised classification to 19 functional groups was used. The figures in the parenthesis represent the number of all *E. coli* genes belonging to the pertinent category and the percentage of differentially expressed genes from this whole group.
[b]The column illustrates the proportion of each gene-specific mRNA from the whole expressed mRNA. In order to facilitate the comparison, the percentage values were multiplied by one hundred (100). The bottom of the column gives the real percentage of the sum mRNA in the wild-type.

patterns were revealed at the 3-fold sensitivity level used in this investigation. We emphasize the importance of careful assessment of the precision of the method as well as individual scrutiny of every gene accepted in the final expression analysis. The cDNA arrays with at least two replicas for each gene provide a very versatile tool to determine the pertinent assay precision. The current results demonstrated a good interassay precision as evaluated by using different *E. coli* substrains.

### Acknowledgement

This work was supported by grants from the Medical Research Fund of Tampere University Hospital (JAV2000, CAS2000), and the Finnish Cancer Organization (JAV1999). We are very grateful to the Sigma-Genosys laboratory personnel who performed the membrane hybridization analyses and provided the comparison data.

### References

Arfin SM, Long AD, *et al.* 2000. Global gene expression profiling in *Escherichia coli* K12: The effects of integration host factor. *J Biol Chem* **275**: 29672–29684.

Blattner FR, Plunkett G 3rd, Bloch CA, *et al.* 1997. The complete genome sequence of *Escherichia coli* K-12. *Science* **277**: 1453–1474.

Reed HR, Henry RJ. 1974. Accuracy, Precision, Quality Control, and Miscellaneous Statistics. In *Clinical Chemistry Principles and Technics*, Henry RJ, Cannon DC, Winkelman JW (eds). Harper & Row Inc.: Hagerstown; 287–341

Salmelin C, Hovinen J, Vilpo J. 2000. Polymyxin permeabilization as a tool to investigate cytotoxicity of therapeutic aromatic alkylators in DNA repair-deficient *Escherichia coli* strains. *Mutat Res* **467**: 129–138.

Copyright © 2002 John Wiley & Sons, Ltd.

Sambrook J, Fritsch E, Maniatis T. 1989. *Molecular Cloning. A laboratory manual*. Cold Spring Harbor Laboratory Press.

Tao H, Bausch C, Richmond C, Blattner FR, Conway T. 1999. Functional genomics: expression analysis of *Escherichia coli* growing on minimal and rich media. *J Bacteriol* **181**: 6425–6440.

Tseng GC, Oh MK, Rohlin L, Liao JC, Wong WH. 2001. Issues in cDNA microarray analysis: quality filtering, channel normalization, models of variations and assessment of gene effects. *Nucleic Acids Res* **29**: 2549–2557.

Sigma-Genosys URL: **http://www.genosys.com**

Copyright © 2002 John Wiley & Sons, Ltd.
